# A Small Molecule Inhibitor of VE-PTP Activates Tie2 in Schlemm's Canal Increasing Outflow Facility and Reducing Intraocular Pressure

**DOI:** 10.1167/iovs.61.14.12

**Published:** 2020-12-14

**Authors:** Guorong Li, Astrid F. Nottebaum, Mitchell Brigell, Iris D. Navarro, Ute Ipe, Sarthak Mishra, Maria Gomez-Caraballo, Heather Schmitt, Brandi Soldo, Steve Pakola, Barbara Withers, Kevin G. Peters, Dietmar Vestweber, W. Daniel Stamer

**Affiliations:** 1 Department of Ophthalmology, Department of Biomedical Engineering, Duke University, Durham, North Carolina, United States; 2 Max Planck Institute of Molecular Biomedicine, Muenster, Germany; 3 Aerpio Pharmaceuticals, Inc., Cincinnati, Ohio, United States

**Keywords:** intraocular pressure, trabecular meshwork, glaucoma pharmacology, Schlemm's canal, Tie-2

## Abstract

**Purpose:**

Tyrosine kinase with immunoglobulin-like and EGF-like domains 2 (Tie2) activation in Schlemm's canal (SC) endothelium is required for the maintenance of IOP, making the angiopoietin/Tie2 pathway a target for new and potentially disease modifying glaucoma therapies. The goal of the present study was to examine the effects of a Tie2 activator, AKB-9778, on IOP and outflow function.

**Methods:**

AKB-9778 effects on IOP was evaluated in humans, rabbits, and mice. Localization studies of vascular endothelial protein tyrosine phosphatase (VE-PTP), the target of AKB-9778 and a negative regulator of Tie2, were performed in human and mouse eyes. Mechanistic studies were carried out in mice, monitoring AKB-9778 effects on outflow facility, Tie2 phosphorylation, and filtration area of SC.

**Results:**

AKB-9778 lowered IOP in patients treated subcutaneously for diabetic eye disease. In addition to efficacious, dose-dependent IOP lowering in rabbit eyes, topical ocular AKB-9778 increased Tie2 activation in SC endothelium, reduced IOP, and increased outflow facility in mouse eyes. VE-PTP was localized to SC endothelial cells in human and mouse eyes. Mechanistically, AKB-9778 increased the filtration area of SC for aqueous humor efflux in both wild type and in Tie2^+/−^ mice.

**Conclusions:**

This is the first report of IOP lowering in humans with a Tie2 activator and functional demonstration of its action in remodeling SC to increase outflow facility and lower IOP in fully developed mice. Based on these studies, a phase II clinical trial is in progress to advance topical ocular AKB-9778 as a first in class, Tie2 activator for treatment for ocular hypertension and glaucoma.

Open-angle glaucoma (OAG) is a leading cause of blindness worldwide, affecting approximately 45 million people. In the United States, OAG impacts approximately 3 million people, a number that is expected to increase to 7.3 million by 2050.^1^^–^^3^ OAG is characterized by death of retinal ganglion cells, degeneration of the optic nerve and progressive vision loss. Elevated IOP is a primary and modifiable risk factor for OAG. Fortunately, OAG is treatable, and effective reduction in IOP slows or prevents vision loss.^4^^–^^7^ Despite the availability of efficacious IOP-lowering drugs, many patients require multiple agents to control IOP. Unfortunately, current drug combinations often fail to achieve a “target” IOP to minimize disease progression.^8^

The conventional outflow (CO) pathway, consisting of the trabecular meshwork (TM) and Schlemm's canal (SC), regulates IOP within a narrow range in healthy eyes and is the site of increased resistance to aqueous outflow in OAG.^9^^–^^11^ First line therapies for OAG do not target CO, but rather reduce IOP by either decreasing the formation of aqueous humor or increasing outflow through the secondary, unconventional outflow pathway. The failure of first line therapies to modify CO is thought to contribute to continued deterioration of the CO pathway and progressive elevation in IOP over time.^12^^,^^13^ The goal of recent therapies in development is to target the pathology in the CO pathway to improve treatment efficacy, either as monotherapy or in combination with approved glaucoma agents.^14^^–^^17^

Recent data suggest that the angiopoietin (Angpt) – tyrosine kinase with immunoglobulin-like and EGF-like domains 2 (Tie2) pathway is critical in the development and maintenance of the CO pathway. Specifically, Tie2 is expressed and activated in SC endothelial cells both during development and after a mature SC lumen has formed in mice.^18^^–^^20^ Disruption of the Tie2 pathway in mice, by conditional knockout of Tie2 or its ligands (Angpt1 and Angpt2) early in postnatal development, results in failure of SC formation, which is associated with increased IOP and optic nerve pathology resembling human congenital glaucoma.^19^^–^^21^ Developmental SC defects in Tie 2 heterozygote mice can be partially rescued by mice expressing only one allele of vascular endothelial protein tyrosine phosphatase (VE-PTP); but interference with VE-PTP expression or function in fully developed mice was not reported.^22^ Supporting these mouse studies, human genetic studies show that both Tie2 and Angpt1 loss-of-function variants associate with risk of congenital glaucoma, and single-nucleotide polymorphisms in the Angpt1 promoter region significantly associate with ocular hypertension and OAG risk.^21^^,^^23^^–^^26^ Taken together, these data support activation of the Angpt/Tie2 pathway in SC as a novel therapeutic target for OAG.

AKB-9778 (razuprotafib; Aerpio Pharmaceuticals, Inc.) is a small molecule that activates Tie2 by inhibiting the intracellular catalytic domain of VE-PTP.^27^^–^^29^ AKB-9778 has been in development as a novel subcutaneous therapy for the treatment of diabetic eye disease.^30^^–^^32^ In two consecutive clinical trials in ocular normotensive human patients with diabetic eye disease, subcutaneous administration of AKB-9778 significantly lowered IOP. To investigate the mechanism responsible for IOP-lowering in humans, preclinical studies in two different animal models show that activation of the Tie2 pathway by AKB-9778 reduces IOP by increasing CO facility. Importantly, VE-PTP uniquely localizes to SC in the CO pathway, and topical ocular administration of AKB-9778 activates Tie2 in SC endothelial cells and increases the luminal size of SC, providing the first demonstration of a potential therapy for OAG that selectively targets and modifies SC in the CO pathway.

## Materials and Methods

### Human Clinical IOP Studies

The TIME-2 clinical trial (NCT02050828), was a phase IIa, double-masked, parallel-group trial designed to assess the effects of subcutaneous AKB-9778 alone or as an adjunct to intravitreal Lucentis, in which 144 patients with diabetic retinopathy complicated by center-involved diabetic macular edema (DME) were randomized to 3 months of one of 3 treatments: AKB-9778 (15 mg) subcutaneous twice daily (BID) plus sham monthly intravitreal injection (IVT); AKB-9778 subcutaneous BID plus Lucentis (0.3 mg) monthly IVT; and placebo subcutaneous BID + Lucentis IVT. Details of the design, methods, baseline demographic data, and DME results of this study have been previously published.^32^ At baseline, mean age was 61.3 ± 1.3 years; 59% of subjects were men; 82.6% had type 2 diabetes; mean HBA1c was 7.5% ± 1.3%; mean best corrected visual acuity was 60.2 ETDRS letters (Snellen 20/63); and mean IOP was 15.7 mm Hg ± 3.4. Relevant to the current analysis, ophthalmic safety assessments were performed at baseline and monthly during the 3-month treatment period including a single IOP measurement by applanation tonometry in both the study eye and the fellow eye at each time point. IOP measurements were conducted by qualified site personnel who had received protocol-specific training. Sites were encouraged to have the same operator conduct all IOP measurements for a given subject. By protocol, all patients had baseline IOP < 24 mm Hg (i.e. were ocular normotensive).

In the prespecified safety analysis, change from baseline IOP was analyzed using a *t*-test (null hypothesis 0 change from baseline). The level of significance was set at 0.05. A post hoc analysis of the subject level IOP measure was the change from baseline in the average over both eyes within a subject for each time point. Statistical testing of each time point was completed using ANCOVA with baseline IOP (averaged over both eyes within a subject) as covariate; additionally, statistical testing averaging across all time points was completed using a mixed-model repeated measures ANCOVA. The level of significance was set at 0.05.

The TIME2b clinical trial was a phase II, randomized placebo-controlled, double-masked clinical trial to assess the safety and efficacy of subcutaneously administered AKB-9778 15 mg once daily or 15 mg twice daily monotherapy for 48 weeks in patients with moderate to severe nonproliferative diabetic retinopathy (NPDR). The study was conducted at 55 sites in the United States. The study was carried out with institutional review board approval. Informed consent for the research was obtained from all subjects and the study complied with the Declaration of Helsinki and the Health Insurance Portability and Accessibility Act. The study was registered at www.clinicaltrials.gov under the identifier NCT03197870, June 23, 2017.

Eligible subjects were aged 18 to 80 years with moderate to severe NPDR. The major inclusion criteria for the qualified eyes included Early Treatment Diabetic Retinopathy Study (ETDRS) Severity Score ≥ 43 and ≤ 53, no evidence of central involved DME and ETDRS ≥ 70 letters using the ETDRS visual acuity charts. Study eye ocular exclusion criteria included uncontrolled glaucoma defined as IOP ≥ 30 mm Hg on maximum IOP reduction therapy.

Subjects were randomized 1:1:1 to either AKB-9778 15 mg once daily (QD), AKB-9778 15 mg twice daily (BID), or placebo BID treatment groups. Subjects self-administered the masked study medication (AKB-9778 or placebo) supplied as sterile pre-filled single-use syringes. Subjects visited the clinical site monthly during the 48-week treatment period. A battery of comprehensive ophthalmic evaluations, including IOP assessments were conducted every 12-weeks with either applanation tonometry or tonopen, and the methodology was consistent throughout the study. IOP measurements were conducted by qualified study personnel who had received protocol-specific training. Sites were encouraged to have the same operator conduct all IOP measurements for a given subject. Only a single measurement was made on each eye. On day 1 and week 24, IOP was measured prior to administration of the morning dose of study medication. On all other days, the time of IOP measurement and relation to the last dose of study medication was not controlled.

IOP data was analyzed using a mixed-model repeated measures ANCOVA, with visit as the repeated measure within subject. The model included change from baseline as the dependent variable. The *P* value from the model is adjusted by baseline IOP.

### Rabbit IOP Study

The rabbit efficacy study was conducted by Absorption Systems (San Diego, CA, USA). Twenty-five female New Zealand White rabbits were administered AKB-9778 for 7 days QD or BID topically (30 µL drop) into both eyes or twice daily subcutaneously, or vehicle twice daily topically into both eyes.

Five dose groups (5 rabbits per group) were tested: vehicle control - 15% HPβCD + 1% dextrose; 1.5% (15 mg/mL) AKB-9778 BID; 4.0% (40 mg/mL) AKB-9778 BID; 4.0% (40 mg/mL) AKB-9778 QD; and 10 mg/kg (40 mg/mL) AKB-9778 administered subcutaneously BID.

IOP measurements were performed at baseline (prior to the start of dosing), and once daily on days 2 to 8. Prior to performing IOP measurements, 1 to 2 drops of a 0.5% proparacaine solution were applied to the eye as a topical anesthetic. IOP measurements were performed with a pneumotonometer 2 hours after AM dosing; the only exceptions were day 8 measurements, which were performed 24 hours after the final dose.

IOP data were analyzed using mixed-design two-factor ANOVA, with time as the within-subjects factor and treatment as the between-subjects factor. IOP values for day 7 and day 8 were analyzed using one-way ANOVA. The change from baseline IOP on day 7 and on day 8 was calculated for each animal, and the resulting values were analyzed using 1-way ANOVA. The area under the curve (AUC) was calculated for all AM IOP measurements throughout the study for each group; AUC values for the different groups were analyzed using 1-way ANOVA. Where appropriate, post hoc testing was performed using Tukey's multiple comparisons tests. The level of significance was set at 0.05.

### Mouse Immunohistochemistry Studies

#### Mice

All experiments involving mice were approved by the Landesamt für Natur, Umwelt und Verbraucherschutz Nordrhein-Westfalen, protocol number 81-02.04.2019.A443. VE-PTP^+/mut^ mice expressing β-Galactosidase under the endogenous VE-PTP promotor were described earlier,^33^ C57BL/6JRj were purchased from Janvier Labs, Tie2^+/−^ hemizygous mice were bred on a C57BL/6JRj background, and originally provided by Daniel Dumont (Toronto).^34^

#### Treatment of Mice With AKB-9778

Mice were anesthetized using Ketamine (125 mg/kg) and Xylazine (12.5 mg/kg). Ten µl of AKB-9778 eye drop solution (4% AKB-9778) or vehicle was applied to each eye. Mice were euthanized 1 hour following dosing, and eyes were processed for immunofluorescence analysis to detect Tie2 phosphorylation.

To analyze the effect of AKB-9778 on SC size, 4-week old wild type (WT) or Tie2^+/−^ mice were treated with 5 µl of AKB-9778 eye drop solution (4% AKB-9778) or vehicle twice daily to each eye for 4 weeks. Prior to application of eye drops, mice were anesthetized using an Isoflurane evaporator. After 4 weeks of treatment, mice were euthanized, and SC area was analyzed by immunofluorescence analysis.

To measure SC area in mouse eyes, 16 20× Z-stacks were captured per mouse from cornea whole mounts with a step size of 0.89 µm and a pinhole of 1 airy unit. Maximum intensity projections of these Z-stacks were prepared, the PECAM-positive area in each 20× image was quantified using the software Fiji (ImageJ) and an average value was obtained for each mouse.

#### Antibodies

The following primary antibodies were used for immunofluorescence staining at the indicated concentrations: VE-PTP (rat-anti-mouse-VE-PTP clone 109.1, 10 µg/mL),^33^ (rabbit-anti-human VE-PTP, hVE-PTP 1-8 antibodies were generated in rabbits against a recombinant form of the extracellular fibronectin type III-like domains 1-8 of human VE-PTP and affinity purified using the antigen), VE-cadherin (rabbit-a-mouse-VE-cadherin, pAB42)^35^ Prox1 (goat-a-human-Prox1, R&D, AF-2727, 2 µg/mL); PECAM-1 (rat-a-mouse PECAM-1 clone 1 G5.1 (3 µg/mL) + clone 5D2.6 (1 µg/mL),^36^ (rabbit polyclonal anti-PECAM-1, Abcam, ab28364); Tie2 (goat-a-mouse-Tie2, R&D, AF762, 5 µg/mL); and phospho-Tie2 (rabbit-a-pY992-Tie2, R&D, AF2720, 10 µg/mL). The following Alexa Fluor labeled secondary antibodies (all from Thermo Fisher Scientific) were used at a concentration of 2 µg/mL: donkey-a-rat-IgG-Alexa-488 (A-21208); donkey-a-goat-IgG-Alexa-568 (A-11057); donkey-a-goat-IgG-Alexa-647 (A-10042); and donkey-a-rabbit-IgG-Alexa-568 (A-21447).

#### Detection of β-Galactosidase Activity

For detection of β-Galactosidase activity enucleated eyes were fixed in 2% paraformaldehyde (PFA) for 15 minutes at room temperature (RT), followed by incubation in X-Gal staining solution (1 mg/mL X-Gal, 5 mM K_4_Fe(CN)_6_, 5 mM K_3_Fe(CN)_6_, and 2 mM MgCl_2_ in PBS) overnight at 37°C. On the next day, corneas were isolated, permeabilized, and blocked in blocking buffer (5% donkey serum, and 0.3% Triton X-100 in PBS) for 1 hour at RT, and incubated with primary antibodies in blocking buffer overnight at 4°C. After 6 washes in washing buffer (0.3% Triton X-100 in PBS) for 15 minutes at RT, corneas were incubated with secondary antibodies in blocking buffer for 4 hours at RT in the dark, followed by 6 washes in washing buffer for 15 minutes at RT before mounting in DAKO fluorescent mounting medium. Analysis was performed using a Zeiss LSM 880 confocal microscope.

#### Immunofluorescence Staining

For immunofluorescence staining of whole-mount cornea preparations, the vasculature of mice was perfused with PBS followed by 1% PFA via the left ventricle. Eyes were enucleated, and eyeballs were fixed in 4% PFA for 30 minutes at RT, followed by incubation in fixative solution (1% PFA, 0.1% Triton X-100, and 0.1% NP-40 in PBS) for 30 minutes at RT. Corneas were isolated from the fixed eyeballs, permeabilized, and blocked in blocking buffer (5% donkey serum, 0.2% BSA, and 0.2% Triton X-100 in PBS) for 1 hour or overnight at RT. Incubation with primary antibodies diluted in blocking buffer was performed overnight at RT. After 6 washes with washing buffer (0.3% Triton X-100 in PBS) for 60 minutes at RT, corneas were incubated with secondary antibodies diluted in blocking buffer overnight at RT, followed by 6 washes in washing buffer for 30 minutes at RT before mounting in DAKO fluorescent mounting medium. Analysis was performed using a Zeiss LSM 880 confocal microscope. For detection of phosphotyrosine, 1 mM sodium orthovanadate was added to all buffers.

For immunofluorescence staining of cryostat sections of eyeballs, the vasculature of mice was perfused with PBS followed by 1% PFA via the left ventricle. Eyes were enucleated, and eyeballs were fixed in 2% PFA for 15 minutes at RT, followed by washing 3 times 5 minutes with PBS, embedded in optimal cutting temperature (OCT) medium (Neg-50, Thermo Scientific), and cut at -16°C. Sections were incubated with 10 µg/mL mAb 109.1 against VE-PTP or 5 µg/mL pAb42 against mouse VE-cadherin, followed by secondary antibodies.

#### Histological Analysis

For histological analysis of adult murine eyes, the vasculature of mice was perfused with PBS followed by 1% PFA via the left ventricle. Eyes were enucleated, and eyeballs were fixed in 2% PFA for 15 minutes at RT, followed by incubation in X-Gal staining solution over night at 37°C. Eyes were post-fixed in 4% PFA for 2 hours at RT, embedded in OCT, and frozen. Frozen blocks were cut into 10 µm sections. Analysis was performed using a Zeiss LSM 880 confocal microscope.

#### Human Immunohistochemistry Studies

Anterior segment wedges from human donor eyes (Supplementary Table S1) were fixed in 4% PFA, immersed in Tissue-Tek OCT compound (25608-930, WVR) and frozen. Sections (10 µm) from tissue blocks containing wedges of human anterior segments were sectioned in sagittal plane, placed on slides, and then incubated in 100% ethanol for 10 minutes and washed 3 times 10 minutes in PBS (10010023, ThermoFisher). Nonspecific antigens in tissue sections were blocked with solution containing 1% BSA (Sigma-Aldrich), 5% normal goat serum (Sigma-Aldrich), and 0.25% Triton X-100 (ThermoFisher) in PBS was for 1 hour at RT. Primary antibodies were diluted in blocking solution and filtered through a 0.22-µm filter prior to overnight incubation at 4°C. Sections were incubated with one of two antibodies: rabbit-anti-human VE-PTP IgGs at a 1:25 dilution or rabbit-anti-CD31 IgGs (Abcam, ab28364) at a 1:25 dilution overnight incubation. Subsequently, slides were washed 3 times for 10 minutes with PBS, and were incubated in goat anti rabbit IgGs conjugated to Alexa Fluor 594 (1:500 dilution, Jackson Laboratories) at RT for 1 hour. After washing with PBS, Vector TrueVIEW Autofluorescence quenching kit (SP-8400, Vector Laboratories) was applied for 2 minutes followed by an additional PBS wash for 10 minutes. Cell nuclei were labeled with diamidino-2-phenylindole (DAPI, 5 µg/mL, Sigma-Aldrich) for 10 minutes at RT. Sections were washed a final time with PBS, were mounted with coverslips using Shandon Immu-Mount (9990414, Fisher Scientific), and then sealed with clear nail polish. Labeling of tissue sections were visualized in merged Z-stacks (10 steps, 1 µm per step) with a Nikon Eclipse confocal microscope. Both experimental and control slides were imaged using identical confocal settings at three different magnifications. For VE-PTP labeling, the 594 channel was pseudo colored (violet) to distinguish from CD31 (PECAM) labeling (red). Images were assembled using Photoshop 2018 (Adobe Systems).

### Mouse IOP and Outflow Studies

Mice were handled in accordance with animal care and use guidelines of Duke University (protocol # A020-16-02) and in compliance with the ARVO Statement for the Use of Animals in Ophthalmic and Vision Research. C57BL/6 (C57) mice were purchased from the Jackson Laboratory (Bar Harbor, Maine, USA), bred/housed in clear cages and kept in housing rooms at 21°C with a 12 hour: 12 hour light-dark cycle. Mice were examined at ages between 4 and 6 months old.

#### IOP Measurements in Mice Treated With AKB-9778

Experiments were conducted to test effects of AKB-9778 on IOP. Eye drops (4% AKB-9778 or vehicle) were administered with study staff masked to treatment. Each animal was randomly assigned to receive one masked treatment in one eye and the other masked treatment in the contralateral eye once daily (QD) for 3 consecutive days. IOPs of both eyes were measured prior to eye drop administration using rebound tonometry (TonoLab) between 11 AM and 1 PM daily following the same procedures described previously.^37^^–^^40^ After collecting IOP measurements on the third day, a final drop of AKB-9778 or placebo was administered and IOP was measured again 2 and 4 hours later. For IOP measurements, mice were anesthetized with ketamine (60 mg/kg) and xylazine (6 mg/kg) and IOP was immediately measured as the mice stopped moving, while drifting into light sleep but before becoming fully anesthetized. Each IOP measurement that was recorded was a result of an average of 6 independent measurements per time point, giving a total of 36 measurements from the same eye.

#### Outflow Facility Measurements

An iPerfusion system was utilized to measure outflow facility in mice. This system is custom designed to simultaneously measure outflow facility in paired mouse eyes having low flow rates (nl/min). After 3 days of treatment with either AKB-9778 or placebo, eyes were enucleated and outflow facility was measured in a masked fashion following established methods described previously.^37^^–^^39^ Briefly, mice were euthanized using isoflurane and freshly enucleated, paired mouse eyes were mounted on two stabilization platforms in temperature-controlled perfusion chambers using a small amount of cyanoacrylate glue (Loctite, Westlake Ohio, USA). The perfusion chambers were filled with prewarmed D-glucose in phosphate-buffered saline (DBG, 5.5 mM) and temperature regulated at 35°C. A glass microneedle was back filled with either vehicle or 10 µM AKB-9778 in a masked manner, matching topical treatments. Microneedles were connected to two parallel perfusion systems and were inserted into the anterior chamber of each eye using micromanipulators visualized using a stereomicroscope.

Both eyes were perfused at 9 mm Hg for 45 to 60 minutes to allow acclimatization and deliver the drug or vehicle to the outflow pathway, followed by 9 sequential pressure steps of 4.5, 6, 7.5, 9, 10.5, 12, 15, 18, and 21 mm Hg. Data were analyzed as described previously.^41^ Briefly, a nonlinear flow-pressure model was used to account for the pressure dependence of outflow facility in mice, and the reference facility was analyzed at a reference pressure of 8 mm Hg (approximates the physiological pressure drop across the CO pathway in living mice). A paired two-tailed *t*-test was used to determine difference in facility between paired eyes was statistically significant.

## Results

### Subcutaneous Administration of AKB-9778 Reduces IOP in Ocular Normotensive Patients With Diabetic Eye Disease

**Figure 1.**
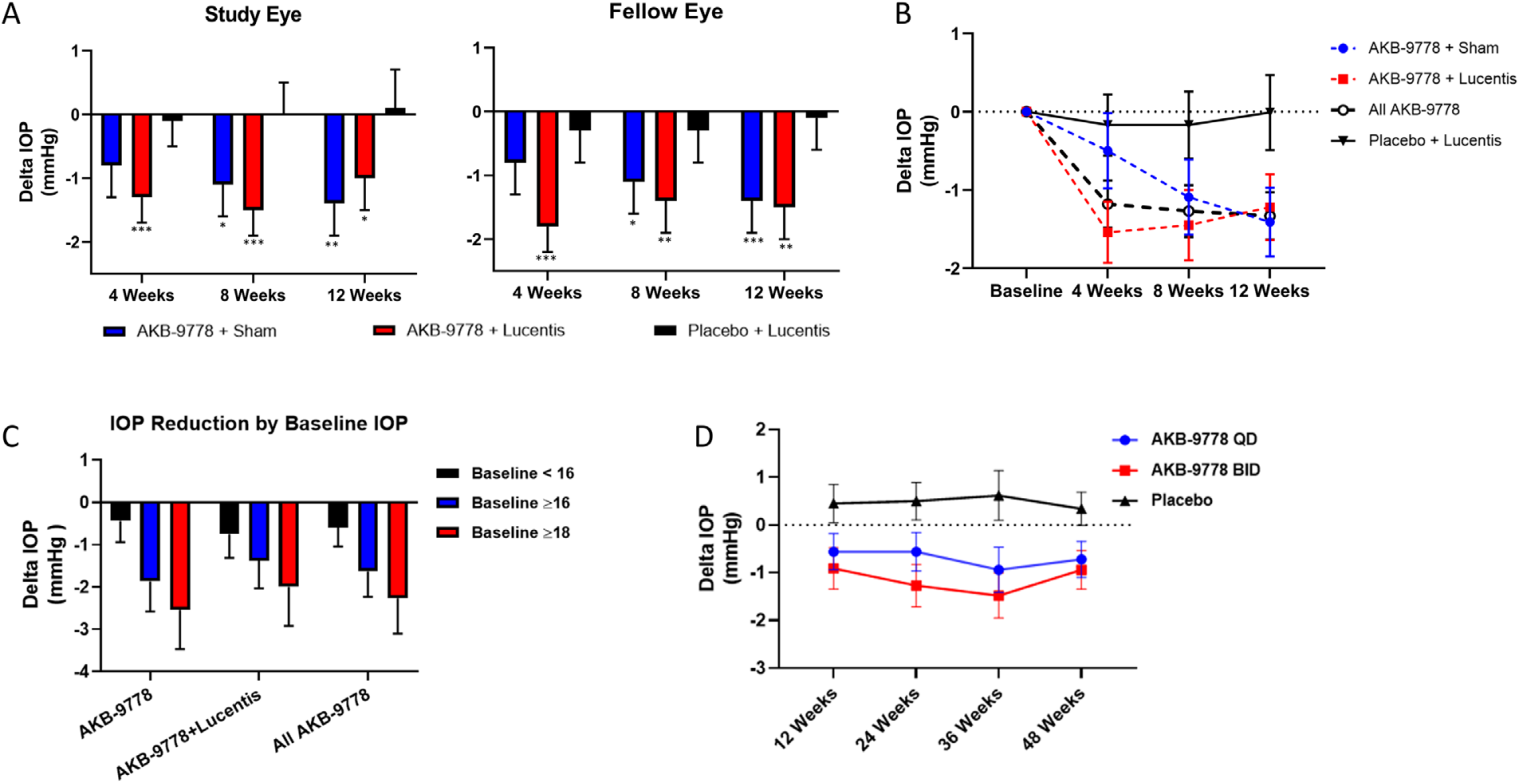
**Effect of subcutaneous AKB-9778 15 mg twice daily on IOP, in two consecutive clinical studies, TIME-2 and TIME-2b, in ocular normotensive patients with diabetic eye disease.** Panels **A** to **C** represent data from the TIME-2 study. Panel (**D**) shows data from the TIME-2b Study. (**A**) In TIME-2, a consistent reduction from baseline (mean change from baseline ± standard error) is seen in both AKB-9778 groups, which was statistically significant in both groups and both eyes (study eye and fellow eye) at every time point with the exception of the AKB-9778 + sham group at 4 weeks. * *P* ≤ 0.05; ** *P* ≤ 0.01; *** *P* ≤ 0.005. (**B**) TIME-2 patient level IOP (average of both eyes, study and fellow eye, within each patient) expressed as mean change from baseline over time in all patients receiving AKB-9778, patients receiving AKB-9778 + sham, patients receiving AKB-9778 + Lucentis, and patients receiving placebo + Lucentis. The IOP differences from baseline (*P* ≤ 0.001 for all AKB-9778 treated groups) and from placebo (*P* = 0.005 for all patients receiving AKB-9778; *P* = 0.017 for AKB-9778 + sham; *P* = 0.013 for AKB-9778 + Lucentis) were statistically significant (MMRM ANCOVA). (**C**) TIME-2 patient level IOP change from baseline as a function of baseline IOP (< 16, ≥ 16, or ≥ 18; placebo adjusted mean ± standard error). Higher baseline IOP was consistently associated with larger IOP reduction consistent with decreased resistance in the pressure-dependent conventional outflow pathway. (**D**) TIME-2b patient level IOP (average of both eyes, within each patient) mean change from baseline over time in all patients receiving AKB-9778 15 mg QD (*blue line*), AKB-9778 15 mg BID (*red line*), and placebo (*grey line*). The IOP change from baseline was statistically significant for the AKB-9778 QD (*P* = 0.041) and BID (*P* < 0.0001) treatment groups, but not for the placebo group (*P* = 0.502). The mean reduction in IOP in the AKB-9778 BID group compared to placebo was 1.15 mm Hg (*P* = 0.0002) and in the AKB-9778 QD group was 0.70 mm Hg compared to placebo (*P* = 0.0552; MMRM ANCOVA).

The TIME-2 study (NCT02050828), a 12-week phase II study in ocular normotensive patients with diabetic retinopathy complicated by DME, was designed to assess the potential benefits of subcutaneous AKB-9778 (15 mg twice daily) on macular edema and visual acuity as a monotherapy (AKB-9778 + sham monthly intravitreal injection) and as an adjunct to monthly intravitreal Lucentis (AKB-9778 + Lucentis monthly intravitreal injection) compared to Lucentis monotherapy (subcutaneous placebo BID + Lucentis monthly intravitreal injection). The overall study design, baseline demographic data, safety data, and DME results (macular edema and visual acuity) have been reported previously.^32^ For patient safety, IOP was assessed at baseline and at monthly intervals over the 12-week study period. The effect on mean IOP change from baseline is shown in Figures 1A, 1B, Supplementary Table S1, and Supplementary Table S2. Subcutaneous administration of AKB-9778 reduced mean IOP by 0.8 to 1.8 mm Hg compared to baseline, which was statistically significant in both eyes at all time points with the exception of the AKB-9778 + sham group at 4 weeks. There was no effect of placebo + Lucentis on IOP compared to baseline (mean change from ‒0.3 to +0.1 mm Hg). Mixed-model ANCOVA analysis, with baseline IOP as the covariate, of IOP data averaged between study and fellow eyes showed a significant reduction in IOP in both AKB-9778-treated groups compared to placebo + Lucentis (AKB-9778 + sham *P* = 0.017, 95% confidence interval [CI] = ‒0.19 to ‒1.83; AKB-9778 + Lucentis *P* = 0.013, 95% CI = ‒0.23 to ‒1.85). There was no statistical difference between the effects of AKB-9778 + placebo and AKB-9778 + Lucentis (*P* = 0.93, 95% CI = 0.78 to ‒0.85) and the interaction of the treatment effect with time was not significant (*P* = 0.881). Importantly, when stratified by baseline IOP, higher baseline IOP was consistently associated with larger IOP reduction, which is consistent with decreased resistance in the pressure-dependent, CO pathway (Fig. 1C).

Importantly, these results were confirmed in a second trial in ocular normotensive patients with diabetic retinopathy, in which significant IOP reduction was observed with subcutaneous AKB-9778 monotherapy, TIME2b (NCT03197870). In the TIME2b trial, subjects were treated with 15 mg AKB-9778 once daily (QD), 15 mg AKB-9778 twice daily (BID), or placebo for 48 weeks. AKB-9778 reduced IOP at all post-baseline time points whereas there was a slight elevation in IOP in the placebo-treated group (Fig. 1D and Supplementary Table S3). There also appears to be a relationship between AKB-9778 dose and IOP lowering, with a larger reduction for the BID dose compared to the QD dose at every time point, ranging from 0.2 to 0.7 mm Hg. A mixed-effect model, repeated measure analysis showed a statistically significant 1.26 mm Hg mean reduction in IOP in the AKB-9778 BID group compared to placebo (*P* < 0.001; 95% CI = ‒0.62 to ‒1.90), whereas the 0.63 mm Hg reduction in the AKB-9778 QD group compared to placebo did not reach statistical significance (*P* = 0.0553, 95% CI = 0.01 to ‒1.27). The 0.63 mm Hg difference between the AKB-9778 QD and AKB-9778 BID group effects on IOP reduction was not statistically significant (*P* = 0.0585, 95% CI = 0.02 to ‒1.27) and there was no interaction with time (*P* = 0.774).

At the 24-week visit, the AM dose was withheld until after the IOP measurement so that drug pharmacokinetics could be assessed. Interestingly, despite a dosing interval of approximately 12 hours or 24 hours for the BID and QD dose groups, respectively, the IOP reduction was similar to other time points in which the AM dose was not withheld, suggesting a sustained IOP lowering effect of AKB-9778.

### Topical Ocular Administration of AKB-9778 Reduces IOP in Ocular Normotensive Rabbits

**Figure 2.**
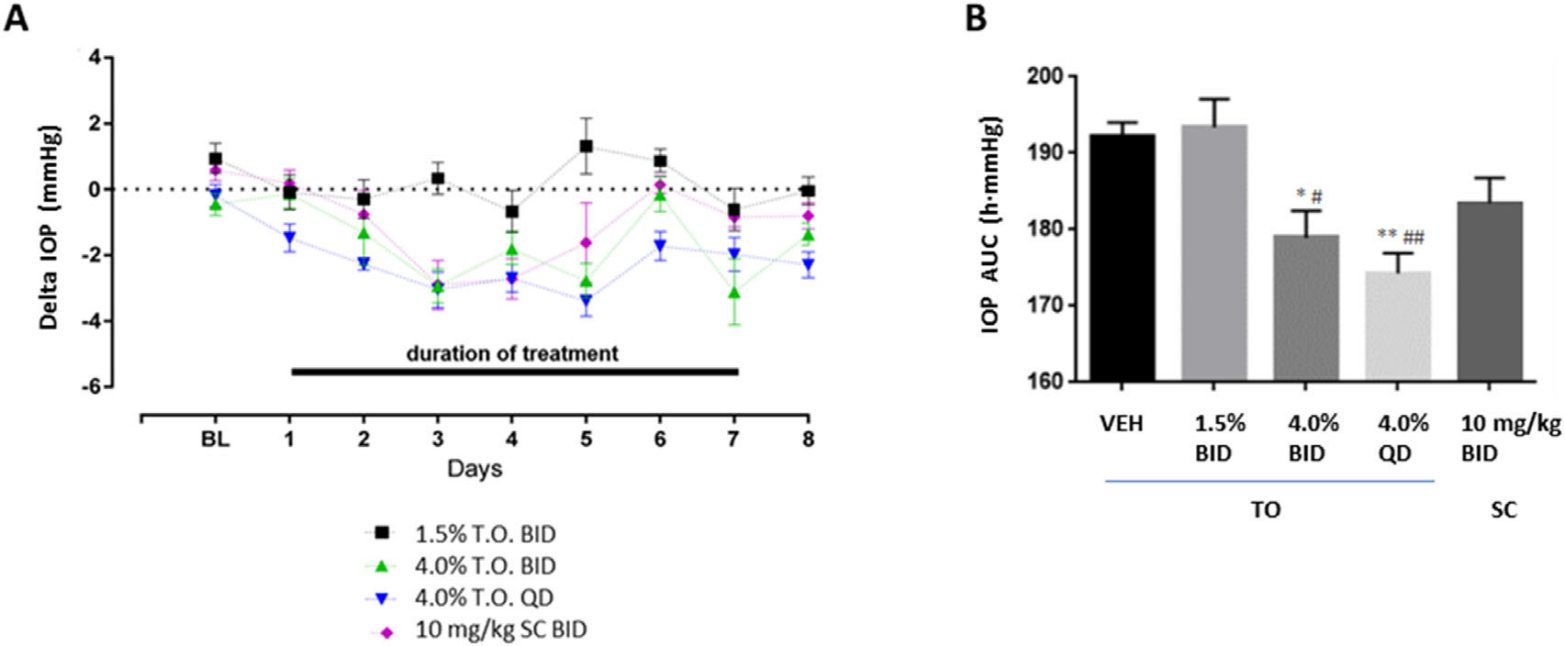
**Effect of Topical ocular administration of AKB-9778 on IOP in ocular normotensive New Zealand White rabbits.** (**A**) Daily mean changes in IOP relative to vehicle control. Decreases in IOP following topical ocular (TO) administration of 4.0% AKB-9778 QD or BID persisted 24 hours postdose (day 8). (**B**) The calculated area under the curve for IOP values (IOP AUC) over 7 days of 4.0% AKB-9778 QD or BID was statistically significantly lower than the IOP AUC values for the vehicle control (vehicle) or 1.5% AKB-9778 BID or 10 mg/kg subcutaneous AKB-9778 BID. n = 5/group; * *P* ≤ 0.05 vs. vehicle; ** *P* ≤ 0.01 vs. vehicle; # *P* ≤ 0.05 vs. AKB 1.5% BID; ## *P* ≤ 0.001 vs. AKB 1.5% BID.

To confirm the IOP reduction observed in patients by systemic administration and explore the potential effects of local administration, AKB-9778 was delivered either by subcutaneous injection (as in the human studies) or by topical drops to eyes of ocular normotensive New Zealand White rabbits and IOP was measured over time (Fig. 2). Topical ocular administration of 4.0% AKB-9778 QD and BID had a significantly larger IOP-lowering effect than topical dosing of the vehicle control or of 1.5% AKB-9778 BID (*P* ≤ 0.001; ANOVA). A steady-state effect on IOP was observed by day 3 following daily topical ocular dosing of 4.0% AKB-9778. In addition, a significant reduction in IOP persisted on day 8, 24 hours after the last dose for the 2 high-dose topical ocular AKB-9778 groups. Although a significant maximum IOP-lowering effect of 10 mg/kg BID subcutaneous injection was observed relative to vehicle control on days 3 and 4 (*P* < 0.01), the effect did not persist in rabbits by the last day of dosing (day 7) and did not differ from vehicle control or 1.5% AKB-9778 BID.

There were no adverse effects of AKB-9778 on local toleration as observed with slit lamp biomicroscopy during the study. McDonald-Shadduck (modified) scoring upon ophthalmologic examination suggested mild conjunctival congestion (score “+1”) observed on day 1 in some rabbits in each group, with a decreased incidence of this finding on day 4. These findings did not persist 24 hours after dosing (day 8).

### Topical Ocular Administration of AKB-9778 Reduces IOP and Increases Outflow Facility in Ocular Normotensive Adult Mice

**Figure 3.**
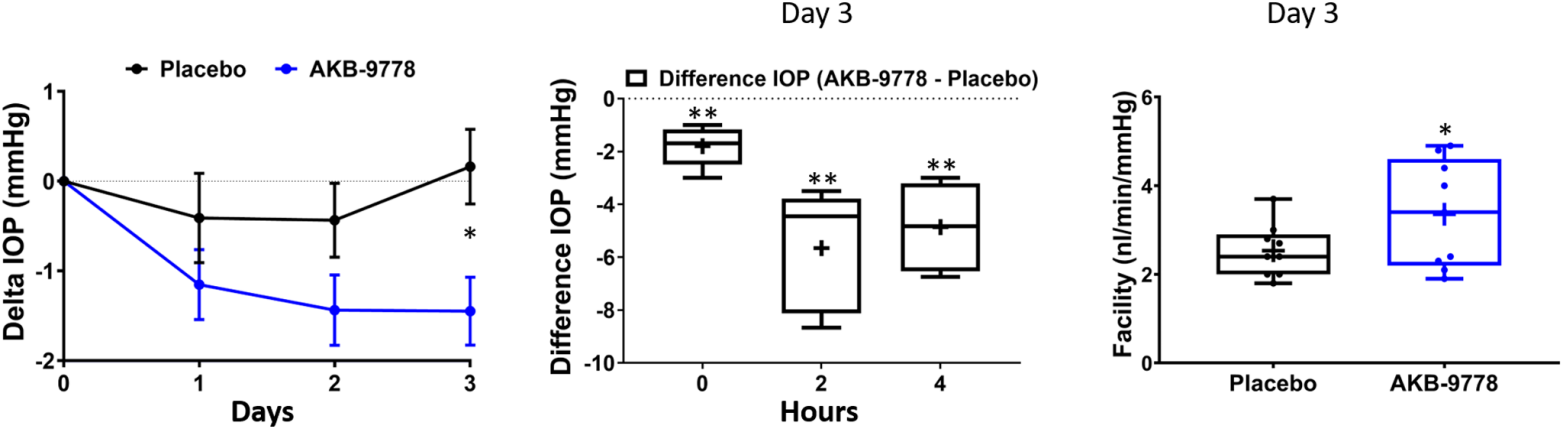
**Effect of AKB-9778 on IOP and outflow facility in ocular normotensive mice.** (**A**) Change in pretreatment IOP (mean ± standard error, n = 14) by comparing pretreatment (day 0) with treatment (placebo or AKB-9778). IOP was measured once per day for 3 consecutive days prior to dosing. (**B**) Time course of IOP lowering after eyes received third daily treatment. Zero hour indicates pretreatment IOP on day 3 (central bar indicates median, the plus sign indicates mean, the range shows minimum to maximum, n = 5) with the additional IOP collections at 2 and 4 hours post eye drop administration. (**C**) Outflow facility (central bar is median, the range shows minimum to maximum, individual data points are indicated, n = 9) of eyes treated with AKB-9778 for 3 days (*P ≤ 0.05, **P ≤ 0.01), comparing drug to placebo-treated contralateral eyes.

In a parallel study, the effect of once daily topical ocular AKB-9778 on predose IOP in naïve eyes of C57 mice was monitored daily for 3 consecutive days. As shown in Figure 3A, AKB-9778 decreased IOP over time relative to IOP on day 0, by ‒0.74 ± 0.43 mm Hg and ‒1.00 ± 0.27 mm Hg when measured just prior to dosing on days 1 and 2, respectively, to a change in IOP of ‒1.6 ± 0.3 mm Hg measured prior to dosing on day 3. Because IOP lowering was maximal and significant on day 3 (*P* = 0.017), IOP was assessed at 2 additional time points on day 3, at 2 and 4 hours after treatment. IOP lowering was more profound at these additional time points, with a significant decrease of approximately 30% (mean ± SE: ‒3.85 ± 1.1 mm Hg at 2 hours and ‒3.1 ± 0.6 mm Hg at 4 hours if compared to both day 0 pretreatment and placebo treatment; ‒5.66 ± 1.0 mm Hg at 2 hours and ‒4.87 ± 0.75 mm Hg at 4 hours if compared to only placebo-treated eyes; *P* < 0.01 by paired Student *t-*test; Fig. 3B).

Due to the known role of the Tie2 pathway in SC and the critical role of the CO pathway in regulating IOP, the effect of AKB-9778 on outflow facility was assessed in mouse eyes. Outflow facility measurements were conducted in mouse eyes that had been dosed for 3 days QD with AKB-9778 and enucleated. As shown in Figure 3C, AKB-9778 significantly increased outflow facility compared to control by 33% (3.4 ± 0.4 vs. 2.5 ± 0.2 nl/min/mm Hg, *P* = 0.047).

### VE-PTP Inhibition Activates Tie2 in Schlemm's Canal of Adult, Normotensive Mice

**Figure 4.**
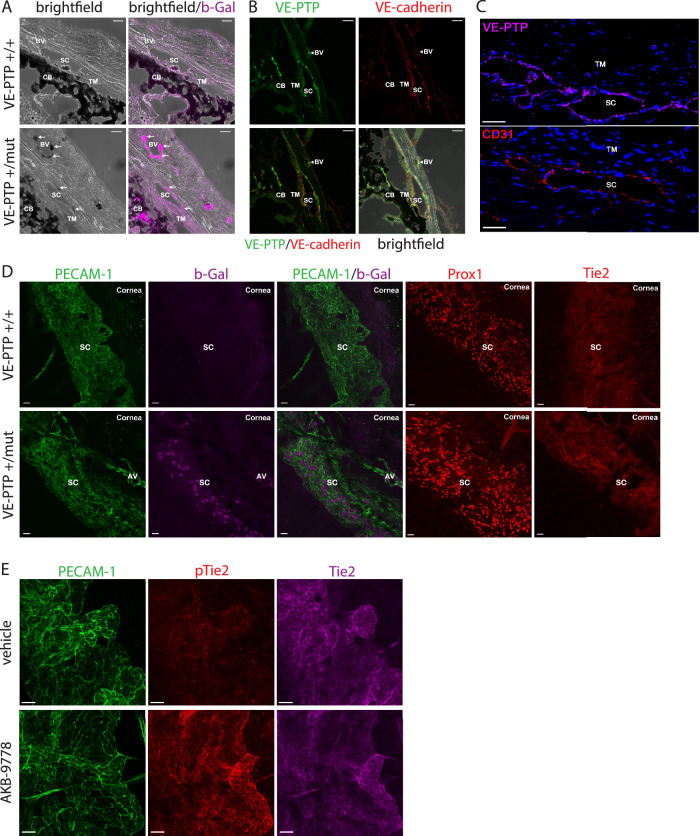
**Expression of VE-PTP and activation of Tie2 by AKB-9778 in SC endothelial cells.** (**A**) Adult murine eyes of either wildtype (VE-PTP^+/+^) or heterozygous knock-in mice expressing β-Galactosidase under the VE-PTP promotor (VE-PTP^+/mut^) were cross-sectioned after X-Gal staining to visualize VE-PTP expression. (**B**) Sagittal sections of adult murine eyes were stained with antibodies specific for VE-PTP (*green*) or VE-cadherin (*red*). (**C**) Top panel shows labelling of conventional outflow tissues from human donor eyes restricted to the endothelial cells of Schlemm's canal (SC) using antibodies specific for VE-PTP (*violet*). Bottom panel shows selective staining of SC with antibodies against the vascular endothelial cell marker, CD31 (*red*). Sections underwent counterstaining with DAPI for cell nuclei localization. TM = trabecular meshwork, magnification bars = 50 µm. (**D**) Whole mount immunofluorescence analysis of adult murine eyes of VE-PTP^+/+^ or VE-PTP^+/mut^ mice. Enucleated eyes were stained as whole mounts with X-gal or with antibodies to detect PECAM-1, Prox-1, and Tie2. The entire thickness of the limbus was visualized by confocal imaging and subsets of optical sections containing SC are shown. (**E**) Whole mount immunofluorescence staining of adult murine eyes topically treated with either AKB-9778 or vehicle for 1 hour and stained for PECAM-1, pY992-Tie2, and total Tie2. The entire thickness of the limbus was visualized by confocal imaging and subsets of optical sections depicting SC are shown. Scale bar = 20 µm; SC = Schlemm's canal; AV = aqueous vein; CB = ciliary body; BV = blood vessel; TM = trabecular meshwork.

To determine potential CO targets of AKB-9778, VE-PTP expression was assessed in anterior eye segments of mice. Previous work showed that VE-PTP protein expression in vivo is limited to endothelial cells of the blood vasculature, but not found in lymphatic endothelium.^33^^,^^42^ VE-PTP expression was assessed in the endothelium of SC in VE-PTP knock-in reporter mice expressing a nuclear-localizing β-galactosidase from the endogenous VE-PTP promotor.^33^ As shown in Figure 4A, β-galactosidase activity was detected in nuclei of SC but not the TM. In parallel, we detected the VE-PTP protein on endothelial cells of SC, but not TM cells, by staining cryostat sections of adult mouse eyes with a monoclonal antibody against VE-PTP (Fig. 4B) and human eye sections with a polyclonal antibody against VE-PTP (Fig. 4C). SC endothelial cells expressed PECAM-1, Prox1, and Tie2 as previously described (Fig. 4D).^20^^,^^43^ To evaluate VE-PTP-mediated signaling, tyrosine phosphorylation and activation of Tie2 in SC following VE-PTP inhibition was evaluated 1 hour following a single topical ocular administration of AKB-9778.^28^ Staining of SC with an antibody specific for tyrosine phosphorylated Tie2 was enhanced following topical ocular administration of AKB-9778 (Fig. 4D), demonstrating Tie2 pathway activation.

### Topical Ocular Administration of AKB-9778 Enhances Filtration Area of Schlemm's Canal in Mice

**Figure 5.**
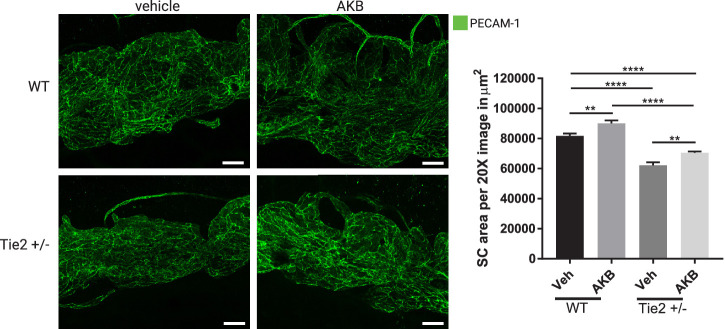
**Topical ocular treatment of WT mice with AKB-9778 increases SC filtration area.** (**A**) Four-week-old C57Bl6 (WT) or Tie2^+/−^ mice were treated twice daily with eyedrops containing AKB-9778 or vehicle for 4 weeks. SC area was analyzed by PECAM-1 immunofluorescence staining of cornea whole mounts. The 20× fields shown represent an area of 146,000 µm^2^. Images were captured as Z stacks with a step size of 0.89 µm and a pinhole of 1 airy unit, and are shown as maximum intensity projections. (**B**) For quantification SC area was measured in sixteen 20× images per condition for 8 WT and 7 Tie2^+/−^ mice; **P ≤ 0.001, ****P ≤ 0.0001.

To determine whether inhibition of VE-PTP by ocular administration of AKB-9778 has an effect on SC morphology in mice, 4-week old WT mice or Tie2^+/‒^ hemizygous mice were treated for 4 weeks BID with eye drops containing AKB-9778 or vehicle. Whole mounts of anterior segments were stained for PECAM-1 and SC areas were measured by ImageJ (Fig. 5A). Significantly, treatment with AKB-9778 enhanced SC filtration area in WT mice by 10.1% (± 3.1%; *P* = 0.0058) and in Tie2^+/−^ mice by 13.3% (± 3.3%; *P* = 0.0017) with topical ocular AKB-9778 treatment compared to vehicle (Fig. 5B).

## Discussion

In two consecutive clinical trials assessing subcutaneous administration of a novel Tie2 activator, AKB-9778, for the treatment of diabetic retinopathy, significant IOP reduction was observed. The IOP-lowering effect of AKB-9778 was subsequently examined in two different animal models to explore its mechanism of action. Topical ocular administration of AKB-9778 in rabbits lowered IOP more than subcutaneous administration in a dose-dependent manner supporting a local ocular effect. In mice, the molecular target of AKB-9778, VE-PTP, was expressed by SC endothelium, and topical ocular administration of AKB-9778 increased Tie2 activation, enhanced SC filtration area, and increased outflow facility, resulting in reduced IOP. Taken together, these data support AKB-9778 as the first glaucoma drug candidate to lower IOP by selectively targeting SC function.

The endothelium of SC is unique, sharing characteristics of both blood and lymphatic vessels.^43^^,^^44^ SC expresses lymphatic endothelial markers such as Prox1, VEGFR3, and the integrin chain α9, but not the typical lymphatic markers, LYVE1 and podoplanin (Table). SC endothelium also expresses blood vascular endothelial markers including Tie2, VEGFR2, PECAM1/CD31, and endomucin. The present study shows the first demonstration of VE-PTP protein expression in SC endothelium, displaying lower expression than neighboring vascular endothelium. These protein data are consistent with differential RNA levels in two recent single cell studies of outflow pathway tissues, emphasizing the hybrid nature of SC.^45^^,^^46^ Other single cell RNAseq studies report the presence of VE-PTP expression in isolated lymphatic cell clusters, however, VE-PTP protein expression in vivo appears distinctly absent from lymphatic endothelial cells in multiple tissues in several studies, including the present one.^33^^,^^42^^,^^47^^–^^49^ One exception appears to be VE-PTP protein expression in cultured human dermal lymphatic endothelial cells.^50^^,^^51^ Perhaps reflecting the hybrid nature of the SC endothelium, VE-PTP was not homogeneously expressed when assessed by reporter gene expression and expression levels appeared generally lower than in vascular endothelial cells. However, expression was found in all regions of SC including its inner wall, and expression of VE-PTP protein was found much more evenly distributed on SC endothelium, consistent with a role in maintenance of SC and regulation of CO facility.

**Table.** Relative Expression of Endothelial Protein Markers in Blood, Schlemm's Canal (SC) and Lymphatic Endothelium^19^^–^^21^^,^^43^^,^^52^^,^^63^

Protein Marker	Blood	SC	Lymphatic
VE-PTP	++	+	−
Tie2	++	+	+
VE-Cadherin	++	+	+
PECAM-1	++	+	+
VEGFR2	++	+	+
VEGFR3	+	+	++
Prox-1	−	+	++
Podoplanin	−	−	++

Results herein provide indirect and direct lines of evidence that AKB-9778 reduces IOP by specifically targeting SC in the CO pathway. Despite the expression of various endothelial markers on cells of the TM, we found no evidence that the TM expressed either Tie2 or VE-PTP, suggesting that the IOP-lowering effect of AKB-9778 was exclusively mediated by SC endothelia.^52^ A second indirect indication was that IOP reduction in rabbits was larger with topical ocular administration than with subcutaneous administration, supporting a local effect of AKB-9778 on IOP regulation. Direct evidence showed that topical administration of AKB-9778 to mouse eyes, rapidly increased Tie2 phosphorylation in SC endothelial cells, enhanced SC area and increased outflow facility following three days of topical ocular dosing, which was consistent with IOP effects of VE-PTP inhibition in both mice and rabbits. Because the system used here functionally isolates the CO pathway, all perfusion media must cross the inner wall of SC where VE-PTP is expressed. Importantly, both mouse and human CO pathway are anatomically alike, having a continuous, circular SC. Similar to AKB-9778 effects on outflow facility in mice, data from the two consecutive human trials show that AKB-9778 is more efficacious in people with higher IOPs, suggesting that AKB-9778 targets the pressure-dependent, CO pathway.

Emerging mouse and human genetic data have established a role for the Tie2 pathway in the development and maintenance of SC. However, the precise molecular and cellular mechanisms of CO facility regulation and IOP lowering have not been fully elucidated. Tie2 activation results in downstream signaling through the AKT, MAP kinase, and eNOS, pathways known to be important for vascular endothelial cell viability and function. It seems likely that these roles are reprised in SC endothelium and contribute to the beneficial effects of Tie2 activation in SC. This is consistent with degeneration of SC that occurs in mice following conditional double knockout of Angpt1 and 2 and with degeneration of SC during aging, both of which were partially rescued with a Tie2 activator, ABTAA.^19^

It was recently reported that the developmental defect of SC size in Tie2^+/−^ mice could be partially compensated by removing one VE-PTP allele in double hemizygous mice.^22^ Although this study indicated a role for VE-PTP during development of SC, no studies were done to assess the role of VE-PTP in the fully developed SC. Moreover, the investigators did not document expression of VE-PTP or enhanced Tie2 activation in SC endothelium and thus an indirect effect of Tie2 activation in other components of the CO tract or systemic vasculature could not be ruled out. In contrast, the findings in the present study validate VE-PTP as a pharmacological target in SC endothelial cells in adult WT mice, emphasizing the role of VE-PTP in SC maintenance and repair.

**Figure 6.**
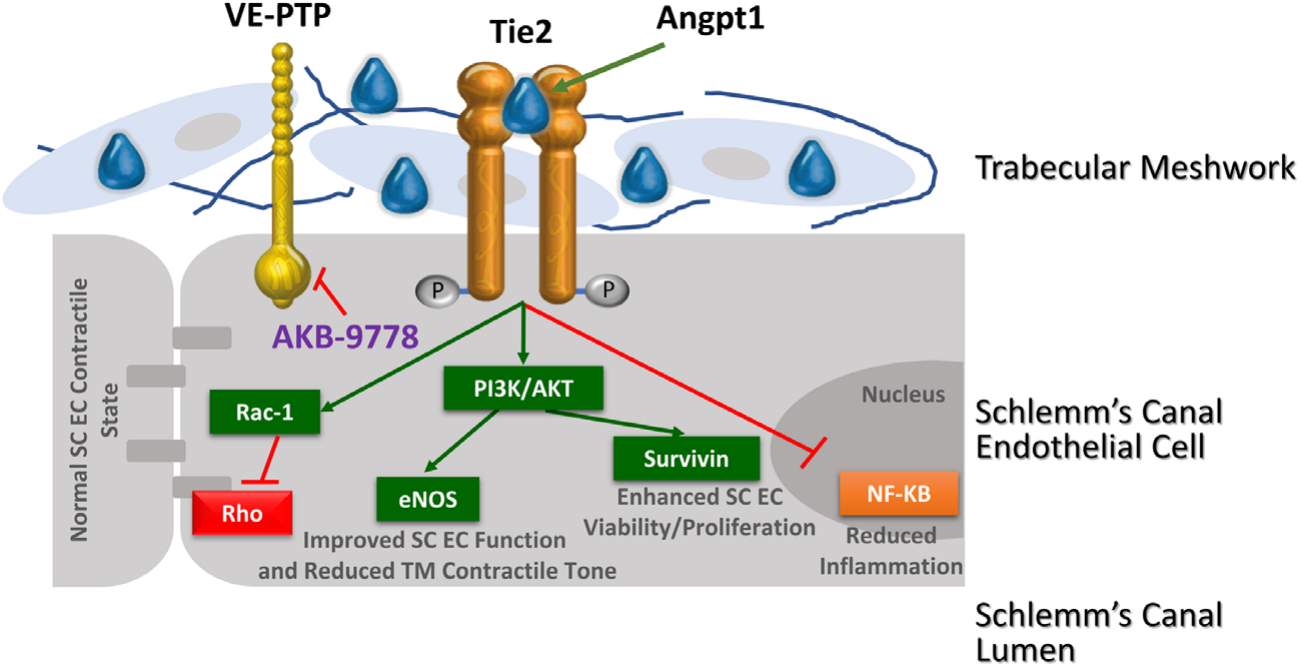
**Potential pleiotrophic effects of AKB-9778 mediated Tie2 activation on restoring normal structure/function of SC.** Tie2 and VE-PTP, the molecular target of AKB-9778 and a critical negative regulator of Tie2 activation, are expressed in Schlemm's canal (SC) endothelial cells (ECs). In the normal SC, Tie2 activation is maintained principally by Angpt-1 produced by cells adjacent to SC endothelial cells including trabecular meshwork cells. Reduced Tie2 signaling has been implicated in the development of congenital glaucoma, ocular hypertension and open angle glaucoma. Tie2 signaling may be important for maintaining both the structural integrity of SC and the normal structure/function relationship between the anterior wall of SC and TM. Tie2 activation with AKB-9778 appears to have both short-term functional effects mediated by eNOS activation and Rho kinase pathway inhibition and longer term anatomic remodeling effects on lumen size of SC, with potential secondary effects on TM. Thus, restoring Tie2 signaling with AKB-9778 represents a novel SC targeted approach to the treatment of OHT/OAG.

Continual replication and replacement of damaged SC cells is critical due to the constant fluctuations in IOP that induce dramatic mechanical stress in the basal to apical direction, forming large outpouchings called “giant vacuoles.”^53^^,^^54^ Interestingly, Tie2 pathway disruption in both knockout and aging mouse eyes reduced the size and number of giant vacuoles characteristic of the inner wall of SC as well as reduced overall SC lumen size. Importantly, SC luminal area is smaller and outflow facility lower in glaucomatous eyes compared to age-matched controls.^55^^–^^57^ Moreover, in Angpt1 conditional KO mice, failure of SC development is associated with marked diminution of TM formation.^21^ Based on these findings and the findings in the current study, Tie2 signaling may be important for maintaining both the structural integrity of SC and the normal structure/function relationship between the anterior wall of SC and juxtacanalicular TM. The interface between SC and juxtacanalicular TM is critical for CO facility and IOP regulation and is known to be the site of increased outflow resistance and pathology in OAG.^58^^,^^59^ Taken together these findings further support Tie2 activation with AKB-9778 as a potential disease modifying approach to treating OAG mediated by the short-term functional effects of eNOS activation and Rho kinase pathway inhibition and the longer-term anatomic remodeling effects on SC and potential secondary effects on juxtacanalicular portion of the trabecular and TM (Fig. 6).

A limitation of the clinical IOP data presented in this report is that the IOP measurements were obtained as safety data in subjects with diabetic retinopathy and normal IOP. As such, the precision of the measurements was probably reduced compared to a typical IOP lowering trial in which measurements are made in triplicate and time of measures are more tightly controlled. It would be expected that a more formal IOP lowering trial in patients with elevated IOP would give additional insight to the true magnitude of the IOP lowering effect of Tie2 activation. A second limitation of the clinical IOP data is that the drug was delivered via subcutaneous administration rather than topical ocular administration.

Notably, similar IOP lowering effects have been observed in ocular normotensive individuals being treated with oral beta-blocker therapy, a class of drugs widely used topically for IOP lowering.^60^^,^^61^ Importantly, these studies suggest that larger IOP lowering effects are expected with topical ocular therapy in patients with OAG/ocular hypertension with higher baseline IOPs.^60^ Consistent with this possibility, there were larger IOP reductions in rabbits with topical ocular versus subcutaneous AKB-9778 (see Fig. 2) and there were larger IOP reductions in patients with ocular normotension with higher baseline IOP (see Fig. 1C). To explore this possibility, a phase II clinical trial is ongoing to assess the IOP lowering effect of topically administered AKB-9778 in patients with OAG/OHT not well controlled with prostaglandins, the current first-line standard of care (NCT04405245). Because AKB-9778 works by increasing CO, it represents an ideal adjuvant therapy to standard of care prostaglandins that reduce IOP primarily via a secondary outflow route known as the uveoscleral or unconventional outflow pathway.^62^

In summary, the present study demonstrates that AKB-9778 lowers IOP in three different species, including humans. Importantly, AKB-9778 is a first-in-class therapeutic agent to increase outflow function and lower IOP by targeting VE-PTP in SC endothelium. Because VE-PTP is only expressed in SC endothelium and not in the TM, this establishes AKB-9778 as the only candidate glaucoma drug to date that selectively interacts with SC. Mechanistically, these results show that AKB-9778 inhibits VE-PTP increasing Tie2 activation resulting in the enlargement of SC filtration area, thereby improving CO function and thus reducing IOP.

## Supplementary Material

Supplement 1
