# Supplementary material for: A Small Molecule Inhibitor of VE-PTP Activates Tie2 in Schlemm's Canal Increasing Outflow Facility and Reducing Intraocular Pressure
Source: Invest Ophthalmol Vis Sci. 2020 Dec 14;61(14):12. doi: 10.1167/iovs.61.14.12 (PMC7735951; doi:10.1167/iovs.61.14.12)
Supplement: Supplement 1 [file iovs-61-14-12_s001.pdf]

Supplementary Tables:

	AKB-9778 + Sham		AKB-9778 + Lucentis		Placebo + Lucentis	
	SE	FE	SE	FE	SE	FE
**Baseline n**	48	48	49	49	47	47
**Baseline IOP mean (SD)**	15.8 (3.69)	15.4 (3.37)	15.9 (3.15)	16.1 (3.18)	15.2 (3.14)	15.8 (6.12)
**Week 4 n**	45	45	48	48	44	44
**Week 1 IOP**	14.8 (3.27)	14.5 (2.81)	14.7 (3.05)	14.4 (3.58)	15.0 (3.50)	15.5 (4.85)
**Change from BL**	-0.8 (3.34)	-0.8 (3.36)	-1.3 (3.04)	-1.8 (2.98)	-0.1 (2.74)	-0.3 (3.49)
**Change BL-Week 4 (p-value)**	0.125	0.118	0.004	<0.001	0.870	0.607
**Week 8 n**	45	45	47	47	44	44
**Week 8 IOP**	14.4 (3.53)	14.3 (3.22)	14.6 (3.54)	14.7 (3.81)	15.0 (3.61)	15.5 (4.52)
**Change from BL**	-1.1 (3.37)	-1.1 (3.58)	-1.5 (3.07)	-1.4 (3.66)	0.0 (3.25)	-0.3 (3.44)
**Change BL Week 8 (p-value)**	0.032	0.052	0.002	0.013	0.926	0.572
**Week 12 n**	45	45	48	48	47	47
**Week 12 IOP**	14.3 (3.56)	14.0 (3.41)	15.1 (3.51)	14.7 (3.39)	15.3 (4.09)	15.7 (5.19)
**Change from BL**	-1.4 (3.16)	-1.4 (3.29)	-1.0 (3.24)	-1.5 (3.49)	0.1 (3.83)	-0.1 (3.62)
**Change BL-Week 12 (p-value)**	0.005	0.005	0.042	0.006	0.879	0.841
BL = baseline; n = number of subjects; SE = study eye; FE = Fellow eye; SD = standard deviation						

Table S1:TIME 2 Intraocular pressure (mmHg) in diabetic subjects with normal IOP.

	AKB-9778 + Sham	AKB-9778 + Lucentis	All AKB-9778	Placebo + Lucentis
Baseline mean IOP (SD)	15.6 (3.33)	16.0 (2.83)	15.8 (3.08)	15.5 (3.82)
Week 4	14.7 (2.88)	14.5 (2.98)	14.6 (2.92)	15.3 (3.49)
Change from BL	-0.8 (3.14)	-1.5 (2.70)	-1.2 (2.93)	-0.2 (2.61)
Week 8	14.4 (3.26)	14.7 (3.38)	14.5 (3.31)	15.3 (3.31)
Change from BL	-1.1 (3.26)	-1.4 (3.10)	-1.3 (3.17)	-0.2 (2.86)
Week 12	14.1 (3.36)	14.9 (3.25)	14.5 (3.30)	15.5 (3.86)
Change from BL	-1.4 (2.98)	-1.2 (2.92)	-1.3 (2.93)	0.0 (3.32)
Change from BL (p-value)	<0.001	<0.001	<0.001	0.56
vs. Placebo (p-value)	0.017	0.013	0.005	
BL = baseline; SD = standard deviation				

Table S2: TIME2 intraocular pressure (mmHg) averaged over both eyes (Study and Fellow eyes) in diabetic subjects with normal IOP.

	AKB-9778 QD	AKB-9778 BID	Placebo
Baseline mean IOP (SD)	15.9 (2.85)	15.6 (2.60)	15.0 (3.14)
Week 12	15.3 (2.76)	14.7 (2.84)	15.4 (2.93)
Change from BL	-0.6 (2.78)	-0.9 (3.11)	0.5 (2.99)
Week 24	15.3 (2.76)	14.3 (2.53)	15.4 (2.92)
Change from BL	-0.6 (2.96)	-1.3 (3.25)	0.5 (2.91)
Week 36	14.9 (3.57)	14.0 (2.66)	15.5 (2.76)
Change from BL	-0.9 (3.36)	-1.5 (3.42)	0.6 (3.85)
Week 48	15.1 (290)	14.6 (2.60)	15.2 (2.81)
Change from BL	-0.7 (2.78)	-0.9 (2.95)	0.3 (2.58)
Change from BL (*p*-value)	0.041	<0.001	0.50
vs. Placebo (*p*-value)	0.055	<0.001	

BL = Baseline; sd = standard deviation

Table S3: TIME2b intraocular pressure (mmHg) averaged over both eyes (Study and Fellow eyes) in diabetic subjects with normal IOP.
